# Prevalence and pattern of multimorbidity in China: a cross-sectional study of 224,142 adults over 60 years old

**DOI:** 10.3389/fpubh.2024.1349418

**Published:** 2024-07-01

**Authors:** Xing Hu, Shugang Li, Zhimin Wei, Dishan Wu, Lingbing Meng, Jianyi Li, Jiapei Xu, Luyao Zhang, Qinan Ma, Hui Li, Xuezhai Zeng, Qiuxia Zhang, Juan Li, Deping Liu

**Affiliations:** ^1^Health Service Department of the Guard Bureau of the Joint Staff Department, Beijing, China; ^2^School of Public Health, Capital Medical University, Beijing, China; ^3^Department of Cardiology, Beijing Hospital, National Center of Gerontology, National Health Commission Institute of Geriatric Medicine, Chinese Academy of Medical Sciences, Beijing, China; ^4^Graduate School, Chinese Academy of Medical Sciences & Peking Union Medical College, Beijing, China; ^5^Department of Cardiology, Beijing Tsinghua Changgung Hospital, School of Clinical Medicine, Tsinghua University, Beijing, China; ^6^China Research Center on Aging, Beijing, China; ^7^Institute of Psychology, Chinese Academy of Sciences, Beijing, China

**Keywords:** the older adult, epidemiology, multimorbidity, risk factors, patterns of multimorbidity

## Abstract

**Aim:**

To examine the prevalence and potential risk factors of multimorbidity among older adult in China. In addition, we investigated the pattern of multimorbidity.

**Methods:**

This study is based on data from the fourth Sample Survey of the Aged Population in Urban and Rural China (SSAPUR) in 2015, a comprehensive survey of individuals aged 60 years or older in China. We calculated baseline data and prevalence rates for comorbidities, stratified by household registration, age, sex, education, exercise, and health insurance. Univariate and multivariate logistic regression analyses were conducted to identify potential risk factors for comorbidities. Furthermore, we determined the prevalence rates for the three most frequent disease combinations.

**Results:**

A total of 215,040 participants were included in our analysis. The prevalence of multimorbidity was 50.5% among the older adult in China. The prevalence rate was slightly higher in rural areas than in urban areas, with rates of 51.5 and 49.6%, respectively (*p* < 0.001). Moreover, the prevalence rate was higher in females than in males, with rates of 55.2 and 45.3%, respectively (*p* < 0.001). Multivariate logistic regression analysis revealed that individuals aged 70–79 years (OR:1.40, 95% CI: 1.38–1.43, *p* < 0.001) and over 80 years (OR:1.41, 95% CI: 1.38–1.45, *p* < 0.001) had a higher prevalence of multimorbidity than those aged 60–69 years. The most prevalent pair of comorbidities was hypertension and osteoarthropathy, with 19.6% of the participants having these two conditions, accounting for 5.4% of the total participants.

**Conclusion:**

Our findings indicate a high prevalence of multimorbidity among the older adult in China. Increased expenditure on preventive health care, popularization of general medicine and popular medical education may be adopted by the Government to cope with the high prevalence of multimorbidity.

## Introduction

Multimorbidity is a prevalent condition, typically defined as the coexistence of two or more chronic diseases, which can result in prolonged hospital stays, increased mortality, elevated medical expenses, and harmful drug reactions for patients (1). Healthcare systems, historically geared up to manage single conditions, are now struggling to cope with the growing complexities of treating patients with multiple chronic health conditions. Notably, the most significant contributors to mortality and disability are diseases associated with the cardiovascular, cerebrovascular, respiratory, and tumors (2–4). Furthermore, the presence of multimorbidity can lead to functional decline, patient frailty, polypharmacy, and reduced quality of life (5). Chronic noncommunicable diseases are linked to aging, which is a leading cause of multimorbidity prevalence (6). A 2019 meta-analysis suggests approximately one-third of adults have multimorbidity globally (7), with rates ranging from 30 to 95% in middle-aged and older adult people (8).

Based on the occurrence of 14 chronic diseases in the 2015 waves of the China Health and Retirement Longitudinal Study (CHARLS), The prevalence of multimorbidity among older adults in China is substantial, estimated at 49.64% (9). However, a review of studies reveals a wide range of estimates, varying from 6.4 to 76.5% among those aged 60 years and older (10). This large variability could be attributed to differences in geography, lifestyle, medical conditions, and data sources across studies. The observed heterogeneity in the prevalence of multimorbidity may also stem from divergent definitions employed or the inclusion criteria specifying the number of diseases to be considered in defining multimorbidity.

In this study, we employed a stratified sampling method to investigate a large Chinese older adult population. Our analysis not only reveals the prevalence of multimorbidity across different age, sex, and education levels, but also identifies common patterns of comorbidities. Our study adopted a stratified sampling method covering all provinces and cities in China. In particular, studies of patterns of multimorbidity hold important implications for policy makers in developing targeted multimorbidity prevention and control strategies.

## Methods

### Data sources

The China Research Center on Aging is a prominent government-affiliated research institution dedicated to aging research. One of its significant initiatives is the Sample Survey of the Aged Population in Urban and Rural China (SSAPUR) project, which began in 2000. This major national condition survey of the older adult in China was followed by longitudinal surveys in 2006 and 2010, the sample size was expanded and resampled in 2015 by Office of the China National Committee on Aging. The 2000 survey included 18,987 observations, the 2006 survey included 18,458 observations, and the 2010 survey included 18,689 observations. We used data from the fourth survey conducted in 2015, which surveyed a vast number of older adult individuals aged ≥60 years in China. The investigation employed a stratified multistage sampling method with probability proportional to size (PPS). This survey collected data from all provinces, autonomous regions, and municipalities, including 466 counties (districts), 1864 townships (sub-districts), and 7,456 village (residential) committees. The SSAPUR is one of China’s largest older adult population databases. For more information on the fourth SSAPUR’s study design and sampling method, please refer to Supplementary materials S1, S2.

The research protocol has been approved by the Ethical Review Committee of Beijing Hospital (No. 2021BJYYEC-294-01) and approved by National Bureau of Statistics (No. [2014] 87). All participants have provided written informed consent.

### Date collection

The data was collected by trained study staff using standardized protocols. At the outset, demographic characteristics including sex, age, education level, exercise and household registration were collected. “Household registration” refers to whether an individual is registered as an agricultural or non-agricultural resident, as recorded in the household register or ascertained by investigators. Education level was categorized as follows: uneducated (no formal education received at any level or from any institution); primary school (completed primary school, dropped out or graduated); junior high school (completed junior high school, dropped out or graduated); high school (completed high school, dropped out or graduated, including general, vocational or secondary professional school); junior college (completed junior college); bachelor’s degree or higher (completed a bachelor’s degree or higher). Exercise refers to all types of intentional physical activity performed for fitness, but excludes housework or farming. Medical insurance refers to the components of China’s medical insurance system, including basic medical insurance for urban workers, basic medical insurance for urban residents, new rural cooperative medical care, and any other medical insurance.

Chronic diseases, including cardiovascular and cerebrovascular diseases (CCVD), cataracts/glaucoma, osteoarthritis, chronic obstructive pulmonary disease (COPD), asthma, malignant tumors, reproductive system diseases (RSD), hypertension, diabetes, and stomach diseases, are self-reported illnesses that have been diagnosed by healthcare professionals before. Multimorbidity is defined as coexistence of two or more chronic diseases in this study.

### Statistical analysis

Prior to analysis, data preparation was undertaken, including removal of missing and ambiguous disease status data. Baseline data and multimorbidity prevalence rates were calculated, and stratified rates were determined according to household registration, age, sex, education, exercise, and health insurance. Analysis was also stratified based on sex, age, and urban/rural location. The prevalence of comorbidities was visually presented, stratified by sex and age categories. Univariate logistic regression analyses were conducted to determine odds ratios and 95% confidence intervals, and significant variables were included in the multivariate regression analysis. A *p*-value of <0.05 was considered statistically significant.

A custom R package was created to calculate the number of cases for each disease combination, of which there were between 45 and 252 possible combinations of 2 to 5 diseases. The number and prevalence of the top three most common disease combinations were also calculated. Statistical analyses were performed with SPSS 24.0 (IBM Corp., Armonk, NY, United States) and R (version 4.1.2).

## Results

In this study, a total of 224,142 cases were examined. Those with missing data were then excluded from the analysis. Specifically, 9,085 cases were removed due to unclear disease status, and 17 individuals were eliminated due to having more than 10 missing independent variables. This resulted in a final sample of 215,040 participants for whom we had access to the relevant variables of interest. The prevalence of 10 self-reported chronic diseases among the older adult population of China was found to be 50.5%. Furthermore, the prevalence was observed to be slightly higher in rural areas (51.5%) compared to urban areas (49.6%). Additionally, the prevalence of chronic diseases was higher in females (55.2%) compared to males (45.3%). Health insurance was found to be associated with higher rates of comorbidities, with a prevalence of 50.6% compared to 42.2% among those without health insurance. Furthermore, our analysis revealed statistically significant differences in the prevalence of comorbidities across different age groups, education levels, and exercise frequency (Table 1).

**Table 1:** Demographics and characteristics of participants.

Characteristics	*N* (percentage%)	Prevalence (%)		
		No disease	1 disease	≥2 disease
Total	215,040 (100)	36,995 (17.2)	69,442 (32.3)	108,603 (50.5)
Household registration				
Urban area	111,938 (52.1)	20,172 (18)	36,324 (32.4)	55,532 (49.6)
Rural area	103,102 (47.9)	16,823 (16.3)	33,208 (32.2)	53,071 (51.5)
Age (years)				
60–69	121,634 (56.6) 64.0 ± 2.8	24,063 (19.8)	41,274 (33.9)	56,297 (46.3)
70–79	63,891 (29.7) 74.1 ± 2.9	8,985 (14.1)	19,446 (30.4)	35,460 (55.5)
≥80	29,515 (13.7) 84.1 ± 3.7	3,947 (13.4)	8,722 (29.6)	16,846 (57.1)
Gender				
Female	112,351 (52.2)	16,204 (14.4)	34,082 (30.3)	62,065 (55.2)
Male	102,689 (47.8)	20,791 (20.2)	35,360 (34.4)	46,538 (45.3)
Education level				
Uneducated	63,104 (29.3)	8,260 (13.1)	18,833 (29.8)	36,011 (57.1)
Primary education	89,752 (41.7)	15,744 (17.5)	29,489 (32.9)	44,519 (49.6)
Junior high school	40,508 (18.8)	8,788 (21.7)	13,863 (34.2)	17,857 (44.1)
High school	15,086 (7.0)	2,967 (19.7)	5,110 (33.9)	7,009 (46.5)
Junior college	4,268 (2.0)	842 (19.7)	1,423 (33.3)	2003 (46.9)
Bachelor degree or above	2,322 (1.1)	394 (17.0)	724 (31.2)	1,204 (51.9)
Exercise (per week)				
Never exercise	105,215 (48.9)	16,978 (16.1)	33,346 (31.7)	54,891 (52.2)
Less than once	9,452 (4.4)	1,585 (16.8)	3,015 (31.9)	4,852 (51.3)
Once or twice	27,582 (12.8)	4,900 (17.8)	9,250 (33.5)	13,432 (48.7)
Three to five times	26,338 (12.2)	4,784 (18.2)	8,673 (32.9)	12,881 (48.9)
Six times and above	46,453 (21.6)	8,748 (18.8)	15,158 (32.6)	22,547 (48.5)
Medical insurance				
No	1960 (0.9)	476 (24.3)	657 (33.5)	827 (42.2)
Yes	213,080 (99.1)	36,519 (17.1)	68,785 (32.3)	107,776 (50.6)

**Figure 1:**
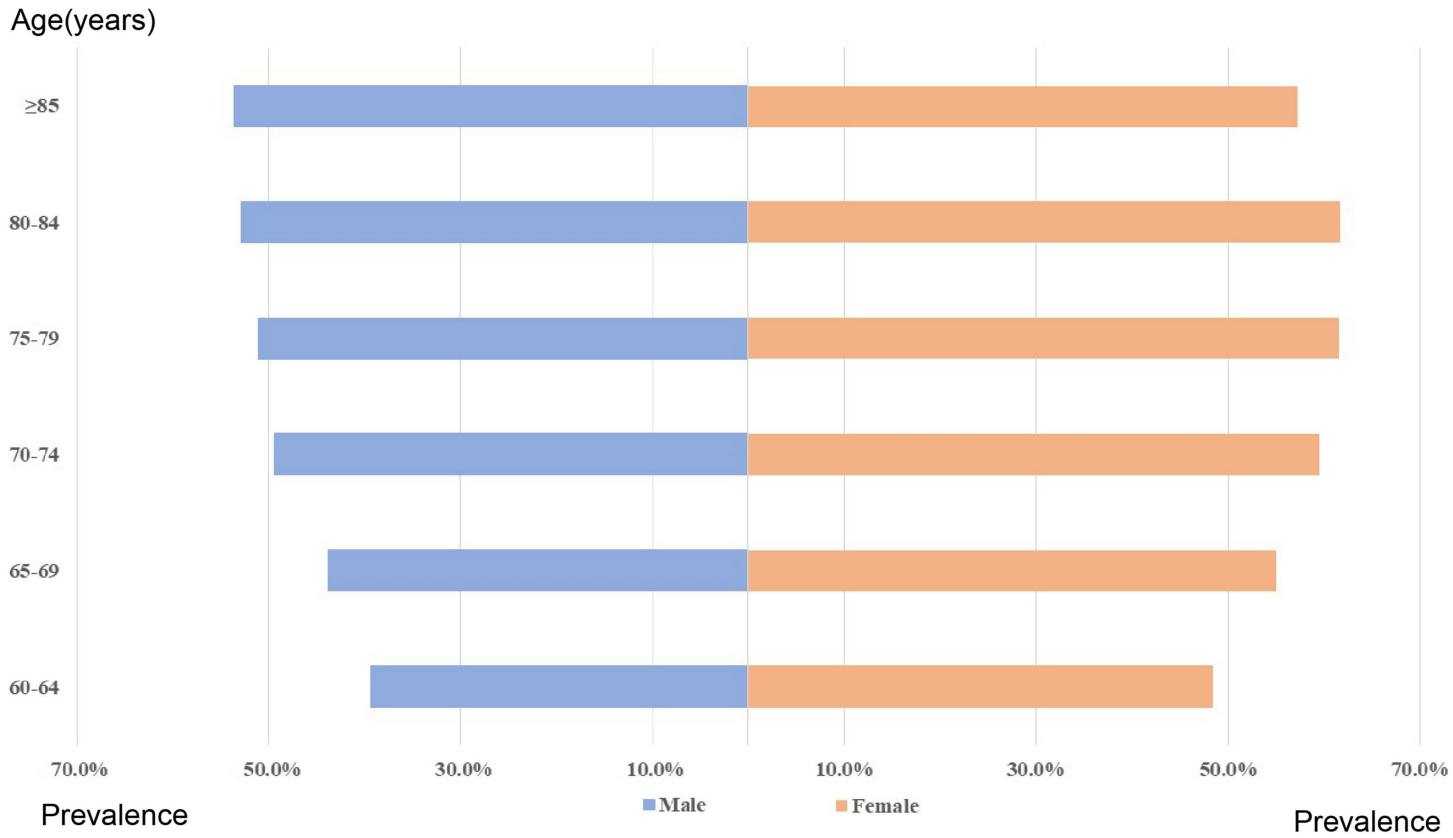
The stratified prevalence of multimorbidity by sex and age.

The prevalence of multimorbidity increased with age, from 39.4% for men aged 60–64 years to 53.6% for men aged 85 years and above (Table 2). Similarly, for women, the prevalence of multimorbidity was 48.4% for those aged 60–64 years, and the highest prevalence was observed in women aged 80–84 years (53.6%) (Figure 1).

**Table 2:** Univariate and multivariate logistic analysis for multimorbidity.

Factor	Univariate		Multivariate	
	OR (95%CI)	*p*-value	OR (95%CI)	*p*-value
Household registration	1.08 (1.06–1.10)	<0.001		
Urban area			1	
Rural area			1.05 (1.03–1.07)	<0.001
Age (years)	1.29 (1.28–1.31)	<0.001		
60–69			1	
70–79			1.40 (1.38–1.43)	<0.001
≥80			1.41 (1.38–1.45)	<0.001
Sex	0.67 (0.66–0.68)	<0.001		
Female			1	
Male			0.72 (0.70–0.73)	<0.001
Education level	0.87 (0.86–0.88)	<0.001		
Uneducated			1	
Primary education			0.88 (0.86–0.90)	<0.001
Junior high school			0.76 (0.74–0.79)	<0.001
High school			0.82 (0.79–0.85)	<0.001
Junior College			0.86 (0.80–0.91)	<0.001
Bachelor degree or above			0.98 (0.90–1.06)	0.595
Exercise (per week)	0.96 (0.96–0.97)	<0.001		
Never exercise			1	
Less than once			1.0 (0.95–1.04)	0.834
Once or twice			0.93 (0.91–0.96)	<0.001
Three to five times			0.97 (0.94–0.99)	0.019
Six times and above			0.98 (0.96–1.00)	0.064
Medical insurance	0.71 (0.65–0.78)	<0.001		
Yes			1	
No			0.68 (0.62–0.75)	<0.001

A regression analysis was conducted to investigate the association between various factors such as household registration, age, sex, education, exercise, and health insurance with the prevalence of multimorbidity. The results showed that all independent variables displayed statistically significant associations. The multivariate analysis indicated that older individuals, specifically those aged 70–79 years (Odd ratio (OR):1.40, 95% confidence interval (CI): 1.38–1.43, *p* < 0.001) and those aged 80 years and above (OR:1.41, 95% CI: 1.38–1.45, *p* < 0.001), had a higher prevalence of multimorbidity compared to those aged 60–69 years. Furthermore, older individuals with higher levels of education and those who exercised once to five times per week were found to have a lower prevalence of multimorbidity (Table 3).

**Table 3:** The prevalence of multimorbidity by sex and age.

Age (years)	Sex	
	Female (%)	Male (%)
60–64	48.4	39.4
65–69	55.0	43.8
70–74	59.5	49.4
75–79	61.5	51.1
80–84	61.7	52.9
≥85	57.2	53.6

**Figure 2:**
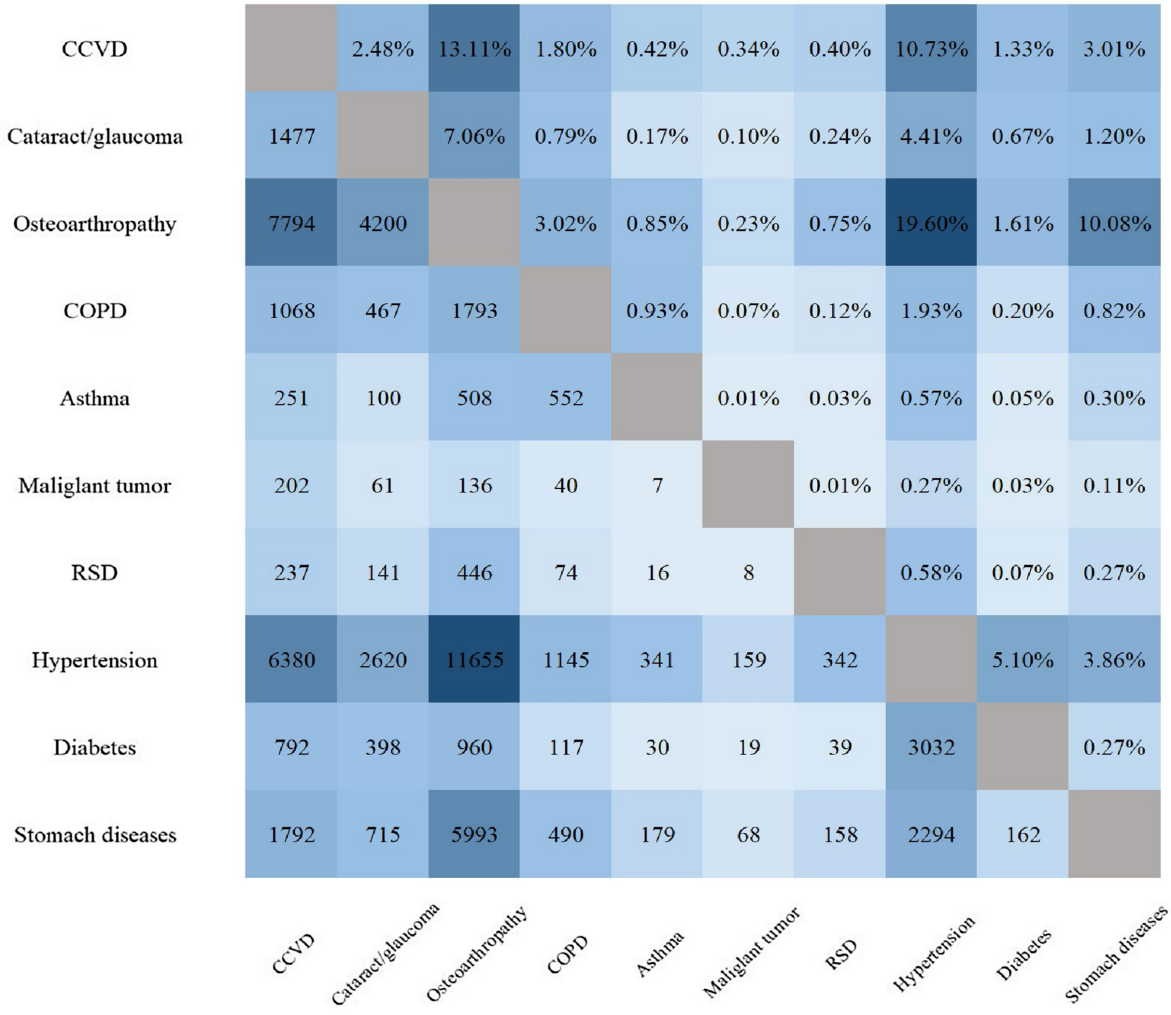
Number and prevalence of 2 diseases. * In the pattern of 2 diseases, the lower left part is the number of patients, and the upper right part is the prevalence accounting for patients with 2 diseases. CCVD, cardiovascular and cerebrovascular diseases; COPD, chronic obstructive pulmonary disease; RSD, reproductive system diseases.

Figure 2 displays the combination patterns and proportions of the two diseases. The most frequent combination was “Hypertension + Osteoarthropathy,” accounting for 19.6% of participants with 2 diseases or 5.4% of the total participants. Additionally, the most common combination of three diseases was “CCVD + Osteoarthropathy + Hypertension” in 2% of the total participants (Table 4).

**Table 4:** The top three multimorbidity situations.

Number of multimorbidity	*N*	Multimorbidity	Prevalence (%)
2 diseases	11,655	Hypertension+ Osteoarthropathy	5.4
	7,794	CCVD+ Osteoarthropathy	5.6
	6,380	CCVD+ Hypertension	3.0
3 diseases	4,228	CCVD+ Osteoarthropathy+ Hypertension	2.0
	3,133	Stomach diseases+ Osteoarthropathy+ Hypertension	1.5
	2793	Cataract/glaucoma+ Osteoarthropathy+ Hypertension	1.3
4 diseases	1,114	CCVD+ Osteoarthropathy+ Hypertension+ Stomach diseases	0.5
	968	CCVD+ Osteoarthropathy+ Hypertension+ Cataract/glaucoma	0.5
	967	Osteoarthropathy+ Hypertension+ Cataract/glaucoma+ Stomach diseases	0.4
5 diseases	337	CCVD+ Osteoarthropathy+ Hypertension+ Cataract/glaucoma+ Stomach diseases	0.2
	248	Osteoarthropathy+ Hypertension+ Cataract/glaucoma+ Stomach diseases+ COPD	0.1
	224	Osteoarthropathy+ Hypertension+ Cataract/glaucoma+ Stomach diseases+ Diabetes	0.1

*CCVD, cardiovascular and cerebrovascular diseases; COPD, chronic obstructive pulmonary disease.

## Discussion

The current study examined the prevalence of self-reported chronic diseases among older adult Chinese individuals, revealing a rate of 50.5%. These diseases spanned numerous systems, including cardiovascular, respiratory, endocrine, digestive, skeletal, and malignant tumor. Notably, two large-scale studies (0.5 million and 2 million participants), conducted by Fanjuningl and Wang X et al., demonstrated multimorbidity prevalence rates of 31.8 and 81.3%, respectively, in individuals aged 60 and over (11, 12). While different populations and combinations of diseases may result in varying prevalence estimates, the accuracy of the questionnaire is an important factor that contributes to these discrepancies (13). Regardless, comorbidities demonstrate high prevalence, significant health economic consumption, and an increased risk of mortality, particularly with respect to concurrent CCVD and RSD (11).

The prevalence of multimorbidity increases with age. Education and exercise may serve as potential protective factors for comorbidities. There is a positive correlation between higher education levels and income, leading to an increased focus on health, comfortable living environments, and access to healthcare. Though the prevalence rates of conditions such as diabetes are higher among highly educated individuals (14), most chronic diseases are less prevalent. Regular exercise is also acknowledged as a healthy habit.

The prevalence of comorbidities in rural areas of China is slightly higher than that in urban areas. This can be attributed to osteoarthropathy caused by manual labor and a higher incidence of COPD or other RSD in rural areas. Additionally, relatively lower economic conditions and fewer healthcare visits can cause chronic disease progression and underestimation of prevalence. A recent study found that individuals living in economically disadvantaged areas develop multiple health conditions 10–15 years earlier than those in more affluent regions (15). It is noteworthy that Chinese government’s rural revitalization program and new rural cooperative medical system have played a significant role in reducing the healthcare disparity between rural and urban areas. The gaps in chronic disease prevalence between rural and urban areas are anticipated to reduce further in the future.

The prevalence of comorbidities was significantly higher in females as compared to males.

A study found that women had a higher prevalence of multimorbidity than men across all age cohorts. Multiple studies have suggested that females may pose a risk factor for the development of comorbidities (8, 16). This difference in prevalence between sex need further investigation.

The study revealed that the most prevalent combination of two comorbidities was “Hypertension+ Osteoarthropathy,” followed by “CCVD+ Osteoarthropathy and CCVD+ Hypertension.” Diseases that Occurred at high frequency in the three-, four-, and five-disease combinations were stomach diseases, cataract/glaucoma, COPD, and diabetes. Kirchberger et al. studied 4,127 participants and found that diabetes, hypertension, stroke, and heart disease (metabolome multimordiopathy combination) had a higher occurrence in the older adult (17). In this investigation, hypertension, CCVD, and diabetes were categorized as metabolic disorders. Hypertension, hyperlipidemia, and diabetes are renowned risk factors for atherosclerosis (18). Large-artery atherosclerotic plaque rupture leads to cardiovascular and cerebrovascular incidents (19). Once CCVD ensues, it typically causes labor loss and a surge in treatment expenses. Metabolic conditions often require long-term use of medications to control blood pressure, lower cholesterol, and manage blood sugar levels. Based on the results of the study, CCVD manifested twice in the two disease combinations, more than hypertension and diabetes. This implies that there may be numerous older adult individuals who are unaware that they have hypertension, diabetes, or hyperlipidemia until they experience CCVD symptoms. Therefore, it is vital to promote blood pressure, blood lipid, and blood glucose management and decrease the cost of fundamental drugs for metabolic diseases for the older adult in China. Considering health economics, these actions can prevent the high cost of CCVD and retain the social workforce, which is of paramount significance.

Several research studies have recognized hearing impairment, eye diseases, and joint diseases as degenerative diseases (20). In this study, Osteoarthropathy and Cataract/glaucoma were also categorized as degenerative diseases. The onset of degenerative diseases is traced back to aging (21). Aging has been found to promote the development of proliferative lesions, including cancer (22). Malignant tumors were not observed among the top three combinations of comorbidities consisting of 2–5 diseases in this investigation. This may be due to their significant impact on patient survival. Meanwhile, COPD and stomach disease were frequent in the multimorbidity group. The number of COPD cases has significantly risen from 32.4 million in 1990 to 54.8 million in 2013 (23), making it a prevailing disease that results in working days lost and mortality, particularly in rural areas and smokers. China has a high incidence of gastritis and gastric cancer, with a high *H. pylori* infection rate. Nearly half of the gastric cancer new cases and deaths caused by *H. pylori* infection worldwide happen in China (24). Therefore, HP screening and elimination together with the popularization of gastrointestinal endoscopy examination need further demonstration.

In recent years, China has made significant progress in the prevention and treatment of multimorbidity. Various strategies and initiatives have been implemented, but there are still areas for improvement.

Firstly, there has been a focus on promoting health education and disease prevention at the community level (25). This includes initiatives aimed at raising awareness about common risk factors for chronic diseases such as hypertension, diabetes, and obesity. Secondly, efforts have been made to improve coordination and integration of care through the establishment of regional healthcare networks (26). These networks facilitate the sharing of medical information and promote collaboration among healthcare providers in different areas. Thirdly, China has prioritized the development of primary healthcare services to ensure early detection and management of chronic diseases (27). The expansion of community health centers and the training of general practitioners have contributed to improved access to healthcare services for individuals with multimorbidity.

Despite these efforts, there are areas where improvements can be made. Firstly, there is a need for better integration of healthcare services across different levels of care, including primary care, secondary care, and tertiary care. This can be achieved through the establishment of formal mechanisms for communication and care coordination between healthcare providers. Secondly, there is a need for more comprehensive and standardized guidelines for managing multimorbidity. These guidelines should provide clear treatment algorithms and recommendations for individuals with multiple chronic conditions (28). Thirdly, there is a need for increased research and data collection on multimorbidity in China in order to better understand the epidemiology and impact of multiple chronic conditions on the population.

### Limitations

The present study has several limitations. Firstly, it did not encompass a comprehensive range of diseases, including renal, intestinal, hepatic, and neurodegenerative conditions. Secondly, the self-reported nature of the diseases among the older adult sample raises concerns about the accuracy of this data. Thirdly, the absence of follow-up data precludes further investigation into the mortality and disability resulting from comorbidities.

## Conclusion

This investigation aimed to document the prevalence and co-occurrence of multimorbidity in Chinese older adult populations across age, sex, and urban–rural residency. In addition, by examining these findings alongside existing literature, this study further explored multimorbidity profiles involving metabolic and degenerative diseases. The implications of this research are substantial, with relevance to both health policymaking and promotion. China has made significant progress in preventing and treating multimorbidity in recent years, but there is still room for improvement. By improving care coordination, developing comprehensive guidelines, and increasing research efforts, the healthcare system can better support individuals with multiple chronic conditions.

## Data availability statement

The original contributions presented in the study are included in the article/Supplementary material, further inquiries can be directed to the corresponding author.

## Ethics statement

Ethical review and approval was not required for the study on human participants in accordance with the local legislation and institutional requirements. Written informed consent from the patients/participants or patients/participants legal guardian/next of kin was not required to participate in this study in accordance with the national legislation and the institutional requirements.

## Author contributions

XH: Conceptualization, Data curation, Formal analysis, Methodology, Writing – original draft. SL: Methodology, Writing – review & editing. ZW: Data curation, Resources, Writing – review & editing. DW: Investigation, Writing – review & editing. LM: Supervision, Writing – review & editing. JiL: Writing – review & editing. JX: Formal analysis, Writing – review & editing. LZ: Resources, Writing – review & editing. QM: Software, Writing – review & editing. HL: Writing – review & editing. XZ: Funding acquisition, Writing – review & editing. QZ: Data curation, Software, Writing – review & editing. JuL: Data curation, Funding acquisition, Writing – review & editing. DL: Conceptualization, Funding acquisition, Methodology, Project administration, Resources, Writing – review & editing.

## References

[1] Yao SS Cao GY Han L Chen ZS Huang ZT Gong P. Prevalence and patterns of multimorbidity in a nationally representative sample of older Chinese: results from the China health and retirement longitudinal study. J Gerontol A Biol Sci Med Sci. (2020) 75:1974–80. doi: 10.1093/gerona/glz185 31406983

[2] Feigin VL Roth GA Naghavi M Parmar P Krishnamurthi R Chugh S. Global burden of stroke and risk factors in 188 countries, during 1990–2013: a systematic analysis for the global burden of disease study 2013. Lancet Neurol. (2016) 15:913–24. doi: 10.1016/S1474-4422(16)30073-4 27291521

[3] Fitzmaurice C Abate D Abbasi N Abbastabar H Abd-Allah F Abdel-Rahman O. Global, regional, and National Cancer Incidence, mortality, years of life lost, years lived with disability, and disability-adjusted life-years for 29 Cancer groups, 1990 to 2017: a systematic analysis for the global burden of disease study. JAMA Oncol. (2019) 5:1749–68. doi: 10.1001/jamaoncol.2019.2996 31560378 PMC6777271

[4] GBD 2017 DALYs and HALE Collaborators. Global, regional, and national disability-adjusted life-years (DALYs) for 359 diseases and injuries and healthy life expectancy (HALE) for 195 countries and territories, 1990-2017: a systematic analysis for the global burden of disease study 2017. Lancet. (2018) 392:1859–922. doi: 10.1016/S0140-6736(18)32335-3 30415748 PMC6252083

[5] Forman DE Maurer MS Boyd C Brindis R Salive ME Horne FM. Multimorbidity in older adults with cardiovascular disease. J Am Coll Cardiol. (2018) 71:2149–61. doi: 10.1016/j.jacc.2018.03.022 29747836 PMC6028235

[6] Kuzuya M. Era of geriatric medical challenges: multimorbidity among older patients. Geriatr Gerontol Int. (2019) 19:699–704. doi: 10.1111/ggi.13742 31397060

[7] Skou ST Mair FS Fortin M Guthrie B Nunes BP Miranda JJ. Multimorbidity. Nat Rev Dis Primers. (2022) 8:48. doi: 10.1038/s41572-022-00376-4 35835758 PMC7613517

[8] Garin N Koyanagi A Chatterji S Tyrovolas S Olaya B Leonardi M. Global multimorbidity patterns: a cross-sectional, population-based, multi-country study. J Gerontol A Biol Sci Med Sci. (2016) 71:205–14. doi: 10.1093/gerona/glv128 26419978 PMC5864156

[9] Guo X Zhao B Chen T Hao B Yang T Xu H. Multimorbidity in the elderly in China based on the China health and retirement longitudinal study. PLoS One. (2021) 16:e0255908. doi: 10.1371/journal.pone.0255908 34352011 PMC8341534

[10] Hu X Huang J Lv Y Li G Peng X. Status of prevalence study on multimorbidity of chronic disease in China: systematic review. Geriatr Gerontol Int. (2015) 15:1–10. doi: 10.1111/ggi.12340 25163532

[11] Fan J Sun Z Yu C Guo Y Pei P Yang L. Multimorbidity patterns and association with mortality in 0.5 million Chinese adults. Chin Med J. (2022) 135:648–57. doi: 10.1097/CM9.0000000000001985, PMID: 35191418 PMC9276333

[12] Wang X Yao S Wang M Cao G Chen Z Huang Z. Multimorbidity among two million adults in China. Int J Environ Res Public Health. (2020) 17:17. doi: 10.3390/ijerph17103395 PMC7277827 32414117

[13] Chua YP Xie Y Lee P Lee ES. Definitions and prevalence of multimorbidity in large database studies: a scoping review. Int J Environ Res Public Health. (2021) 18:1673. doi: 10.3390/ijerph18041673 33572441 PMC7916224

[14] Hu X Meng L Wei Z Xu H Li J Li Y. Prevalence and potential risk factors of self-reported diabetes among elderly people in China: a national cross-sectional study of 224,142 adults. Front Public Health. (2022) 10:1051445. doi: 10.3389/fpubh.2022.1051445 36620236 PMC9811661

[15] Barnett K Mercer SW Norbury M Watt G Wyke S Guthrie B. Epidemiology of multimorbidity and implications for health care, research, and medical education: a cross-sectional study. Lancet. (2012) 380:37–43. doi: 10.1016/S0140-6736(12)60240-2 22579043

[16] Gu J Chao J Chen W Xu H Wu Z Chen H. Multimorbidity in the community-dwelling elderly in urban China. Arch Gerontol Geriatr. (2017) 68:62–7. doi: 10.1016/j.archger.2016.09.001 27654809

[17] Kirchberger I Meisinger C Heier M Zimmermann AK Thorand B Autenrieth CS. Patterns of multimorbidity in the aged population. Results from the KORA-age study. PLoS One. (2012) 7:e30556. doi: 10.1371/journal.pone.0030556 22291986 PMC3264590

[18] Shen C Ge J. Epidemic of cardiovascular disease in China: current perspective and prospects for the future. Circulation. (2018) 138:342–4. doi: 10.1161/CIRCULATIONAHA.118.033484 30571361

[19] Zhang S Liu Y Cao Y Zhang S Sun J Wang Y. Targeting the microenvironment of vulnerable atherosclerotic plaques: an emerging diagnosis and therapy strategy for atherosclerosis. Adv Mater. (2022) 34:e2110660. doi: 10.1002/adma.202110660 35238081

[20] Wang R Yan Z Liang Y Tan EC Cai C Jiang H. Prevalence and patterns of chronic disease pairs and multimorbidity among older Chinese adults living in a rural area. PLoS One. (2015) 10:e0138521. doi: 10.1371/journal.pone.0138521 26394368 PMC4578976

[21] Zhang L Wu J Zhu Z He Y Fang R. Mitochondrion: a bridge linking aging and degenerative diseases. Life Sci. (2023) 322:121666. doi: 10.1016/j.lfs.2023.121666 37030614

[22] Campisi J. Aging, cellular senescence, and cancer. Annu Rev Physiol. (2013) 75:685–705. doi: 10.1146/annurev-physiol-030212-183653 23140366 PMC4166529

[23] Yin P Wang H Vos T Li Y Liu S Liu Y. A subnational analysis of mortality and prevalence of COPD in China from 1990 to 2013: findings from the global burden of disease study 2013. Chest. (2016) 150:1269–80. doi: 10.1016/j.chest.2016.08.1474 27693597

[24] Hu Y Zhu Y Lu NH. The management of *Helicobacter pylori* infection and prevention and control of gastric cancer in China. Front Cell Infect Microbiol. (2022) 12:1049279. doi: 10.3389/fcimb.2022.1049279 36530421 PMC9751207

[25] Wang C Lang J Xuan L Li X Zhang L. The effect of health literacy and self-management efficacy on the health-related quality of life of hypertensive patients in a western rural area of China: a cross-sectional study. Int J Equity Health. (2017) 16:58. doi: 10.1186/s12939-017-0551-9 28666443 PMC5493849

[26] Liang J Zheng X Chen Z Dai S Xu J Ye H. The experience and challenges of healthcare-reform-driven medical consortia and regional health information Technologies in China: a longitudinal study. Int J Med Inform. (2019) 131:103954. doi: 10.1016/j.ijmedinf.2019.103954 31513943

[27] Wang S Wang X Zhou Y Xu J. Utilization of, satisfaction toward, and challenges for internet-based healthcare services provided by primary health institutions: evidence from China. Front Public Health. (2022) 10:1100634. doi: 10.3389/fpubh.2022.1100634 36743153 PMC9892623

[28] Stevens PE Lamb EJ Levin A. Integrating guidelines, CKD, multimorbidity, and older adults. Am J Kidney Dis. (2015) 65:494–501. doi: 10.1053/j.ajkd.2014.09.024 25483849

